# Prevalence and predictors of susceptibility and future intention to smoke cigarettes among school-going adolescents in Ibadan, Nigeria

**DOI:** 10.11604/pamj.2020.37.230.24174

**Published:** 2020-11-11

**Authors:** Mary Ebelechukwu Osuh, Omotayo Francis Fagbule, Yetunde Damilola Olatunji

**Affiliations:** 1 Department of Periodontology and Community Dentistry, Faculty of Dentistry, College of Medicine, University of Ibadan, Ibadan, Nigeria,; 2 Department of Periodontology and Community Dentistry, University College Hospital, Ibadan, Nigeria,; 3 Cephas Health Research Initiative Incorporated (CEPHAS), Ibadan, Nigeria

**Keywords:** Tobacco, future smoking intention, susceptibility to smoke, adolescents, Ibadan, Nigeria

## Abstract

**Introduction:**

the tobacco control interventions targeted at preventing the initiation of tobacco habits are crucial to effective control of tobacco use among adolescents. An understanding of the predictors of smoking susceptibility and future intention to smoke is important in developing effective intervention programmes. This study, therefore, assessed the prevalence and predictors of susceptibility and future intention to smoke cigarettes among school-going adolescents in Ibadan, Nigeria.

**Methods:**

a cross-sectional study among 830 school-going, non-smoking adolescents, who were randomly selected from 18 secondary schools in Ibadan, Nigeria. Using a self-administered, structured questionnaire, information on socio-demography, tobacco attitudes and habits were collected and analyzed using SPSS version 25.

**Results:**

the prevalence of susceptibility and future intention to smoke cigarette were 25.9% and 6.3%, respectively. Predictors of susceptibility were low social-class (aOR:1.68; 95%CI:1.01-2.80); cigarette sale near schools (aOR:2.04; 95%CI:1.16-3.61); poor attitude (aOR:1.93; 95%CI:1.29-2.89); no harm-perception to smoking (aOR:3.55; 95%CI:2.13-5.92), exposure to secondhand smoke (SHS) (aOR:2.31; 95%CI:1.52-3.50) and perceived safety of short-term smoking (aOR:1.59; 95%CI:1.02-2.44). Predictors of future intention to smoke were: ever-tobacco smoking (aOR:2.05; 95%CI:1.003-4.170); cigarette sale near schools (aOR:1.79; 95%CI:1.09-2.94); poor attitude (aOR:1.95; 95%CI:1.31-2.88), no harm perception to smoking (aOR:3.87; 95%CI:2.38-6.31), exposure to SHS (aOR:2.45; 95%CI:1.64-3.67) and perceived safety of short-term smoking (aOR:1.59; 95%CI:1.05-2.44).

**Conclusion:**

a significant proportion of the population had high susceptibility to smoke as well as high future intention to smoke. Sales of cigarettes near schools, poor attitude and poor perception about the harm from smoking and exposure to SHS were important predictors of both susceptibility and future intention to smoke among respondents.

## Introduction

Tobacco has remained the leading cause of preventable death among both the users and non-users but, who are exposed to the smoke to date [1]. Beyond the overwhelming general health effect of tobacco [2,3], it is a risk factor for numerous dental problems such as various gum diseases and pigmentation, malignancies such as leukoplakia and oral cancer [4-7]. It is also associated with oral clefts, dental caries and eventual tooth loss [5,8]. While some of these adverse effects manifest within a short duration of smoking, others occur after a considerable period, such as among adults who started smoking from since adolescence.

Annually, the tobacco epidemic is sustained by the addition of many adolescents and youths to the population of smokers [9] and more than 2,800 adolescents are initiated daily [10]. Four in five adult smokers started smoking before the age of 18 years and 50% of adult smokers suffer tobacco-related death [11]. While the smoking prevalence is decreasing in High-Income Countries (HICs), the situation is different for Africa; where the prevalence is increasing such that if the current trend of weak implementation of tobacco control legislation continues, smoking prevalence rate may be doubled by 2030 [12,13]. This means that adolescents who are not yet smoking will initiate the habit in the future [14]. This development underscores the need for preventive intervention among the group [15]. Prevention programs should target those susceptible or have the intention to smoke, but who are yet to initiate the habit. Susceptibility is described as a lack of firm commitment not to smoke in the future or when offered a cigarette by close friends [16], while smoking intention is the plan to smoke within a year [17]. Being susceptible to smoking or having the intention to smoke in the future are strong predictors for initiating the habit among adolescents [15,16,18-20]. Being an addictive behaviour, there is a higher chance of success for tobacco intervention programmes that are targeted at preventing tobacco uptake than those targeted at cessation [21]. Hence, the need to pay more attention to this group of adolescents that are currently non-smoking adolescents, but who are susceptible or have the intention to smoke in the future.

In a bid to develop effective intervention programs to target adolescents at the pre-initiation stage of tobacco smoking, vital information such as the prevalence and predictors are required. Studies across Low and Middle-Income Countries (LMICs) have reported a high prevalence of adolescents´ susceptibility to smoke (16% to 49.7%) [21-32], as well as those who have the intention to smoke in the future (11.4%-23.3%) [21,24,33,34]. The type of school (public vs private) has been reported to play a role in determining if a student will be susceptible to tobacco smoking. From a study conducted in Lagos, Nigeria, students who were attending privately-owned schools were five times more likely to be susceptible to smoking when compared to their public school counterparts [35]. Similarly, various factors such as male gender, low socioeconomic status, a close friend and family smoking, poor harm perception of smoking, allowing smoking on the school compound and exposure to tobacco advertisement, have been reported to be associated with being susceptible to smoking and having future smoking intention [20,24,36,37]; however, recent information on susceptibility and intention to smoke tobacco is limited in Nigeria. Furthermore, the available studies reported on adolescents´ susceptibility to cigarette smoking, did not provide information on their future smoking intention [35,37]. Hence, we conducted this study to assess the current prevalence and predictors of susceptibility and future intention to smoke cigarette among school-going adolescents in Ibadan, Nigeria. The result of the study will contribute to the scientific information about the susceptibility and future intention to smoke among adolescents in Ibadan, Nigeria. The findings will also be useful in developing effective interventions aimed at preventing tobacco uptake among adolescents.

## Methods

**Study design and procedure:** this study was a part of a larger survey conducted on tobacco use among school going adolescents in Ibadan, Nigeria. The cross-sectional study design was conducted in 2018 among junior school two to senior school three (JSS 2 - SSS 3) students of secondary schools in Ibadan metropolis, Oyo State, Nigeria with ethical approval of its conduct from the University of Ibadan/University College Hospital Ethics Review Board (UI/EC/18/0243) [38]. Following a detailed explanation of the research conduct and thorough understanding, informed consent was obtained from parents while the participants signed the accent forms. Information sought from participants included: socio-demographics, attitude towards tobacco smoking, harm perception, assessment of smoking susceptibility and intention to smoke.

**Population sample:** a representative sample of 900 school-going adolescents in JSS2 - SSS3 from both public and private secondary schools in Ibadan metropolis were randomly selected. A multistage (3 stages) sampling technique was utilized. In the first stage, three local government areas (LGAs) were randomly selected out of the five LGAs that make up the Ibadan metropolis. The second stage involved the selection of six secondary schools from the three LGAs using simple random sampling, making a total of 18 selected schools. The final stage involved the use of stratified random sampling to select about ten students per class (JSS2 - SSS3) [38].

**Data collection procedure:** a self-administered and structured questionnaire adapted from the standardized questionnaire for Global Youth Tobacco Survey (GYTS) [39] was used for data collection.

### Study measures

**Cigarette smoking status:** the participants´ tobacco smoking status was assessed with two questions: “have you ever smoked a cigarette, even if it is one or two puffs” and “during the past 30 days, on how many days did you smoke cigarettes”. Those who responded negatively to the two questions were classified as “never-smokers” and “former smokers” respectively. Both never- and former-smokers were categorized as current non-smokers [40] and they constituted the study population for this study.

**Tobacco smoking status:** participants smoking status was assessed with the question: “have you ever smoked tobacco products other than cigarettes, even if it is one or two puffs”. Those who chose “yes” and “no” were classified as “ever-tobacco smokers” and “never-tobacco smokers” respectively.

**Smoking susceptibility and future intention to smoke:** the participants´ smoking susceptibility and future intention to smoke were assessed using the following questions: a) if one of your best friends offered you a cigarette, would you take it; b) do you think you will be smoking cigarette in five years from now; c) at any time during the next 12 months, do you think you will smoke cigarette. The possible options were “definitely not”, “probably not”, “probably yes” and “definitely yes.” Anyone who failed to choose “definitely not” in all the three questions was deemed to be susceptible, while those who failed to choose “definitely not” in the third question (question c) were deemed to have future smoking intention [20,24].

**Social class:** the social class of the participants was determined based on the classification by Oyedeji *et al*. (1985) [41]. Different scores were assigned to the educational level and occupation of the respondents´ parents/guardians and the average score per participant was calculated. Following this, the respondents were further categorized into low, middle or high social class based on their scores [41].

**Harm perceptions:** perception about the adverse effect of tobacco smoking and the exposure to SHS was assessed by the question: “do you think the smoke from other people´s tobacco smoking is harmful to you”, “do you think smoking tobacco is harmful to your health” and “do you think it is safe to smoke tobacco for only a year or two as long as you quit after that”. The options were: ‘definitely not´, ‘probably not´, ‘probably yes´ and ‘definitely yes´. The first two options were later merged as “no” and the last two options combined as “yes”.

**Attitude towards tobacco smoking:** a new variable was computed from 16 question items to assess the general attitude of the respondents about tobacco smoking. Some of the questions used were “smoking cigarette helps reduce stress”, “smoking cigarette makes you stronger”, “smoking cigarette makes you confident”, “smoking cigarettes is enjoyable”, “smoking cigarettes makes you more intelligent”, “smoking cigarette is cool”, “smokers are bad people”, “smoking helps you make friend”, “people who smoke cigarettes are more popular”, “girls who smoke cigarettes are more attractive to boys”, “boys who smoke cigarettes are more attractive to girls” and “tobacco companies are very bad”. The options were “I believe this”, “I do not believe this”, and “I do not know”. Depending on the question, those with the right attitude were given a score of “1” and others “0”. Subsequently, those with a total score less than eight were categorized as having a “poor attitude/support tobacco smoking” and those with a score of eight and above were categorized as having “good attitude/against tobacco smoking”.

**Statistical analysis:** data were analyzed using Statistical Package for Social Sciences (SPSS) version 25 [42]. Only the data of respondents with the characteristic of interest (current non-cigarette smokers) was analyzed for this study. Descriptive analysis of the variables was done and presented as mean and standard deviation, while the categorical variables were presented as proportions. Associations between the outcome variables (susceptibility and intention to smoke) and the independent variables were done using Chi-square test and those that yielded statistically significant associations were further subjected to logistic regression modelling. The adjusted Odds Ratios (aOR) and 95% Confidence Interval (95%CI) were reported. All statistical inferences were based on a 5% significance level.

## Results

Of the total of 900 selected study participants, 39 of them declined participation in the research, 861 completed and returned the questionnaires, giving a response rate of 95.6% [38]. Thirty-one participants (3.6%) were current smokers and they were removed from the analysis for this study. Thus, leaving a total of 830 current non-smoking adolescents with a mean age of 14 (± 1.72) years.

**Attitude to cigarette smoking:** overall, the majority (78.6%) had a good attitude (against cigarette smoking), agreed that smoking cigarette is harmful to their health (87.3%), that exposure to secondhand smoke (SHS) was harmful to them (78.1%) and that it is not safe to smoke even for a short period (81.7%) (Table 1).

**Table 1:** socio-demographic variables of the study participants

Variables	Frequency (n=830)	Percent (%)
**Age**	**Mean=14.3**	**S.D=1.7**
Late adolescents 15-19 years	367	44.2
Early adolescents 10-14 years	463	55.8
**Sex**		
Male	426	51.3
Female	404	48.7
**Class level**		
Senior classes	468	56.4
Junior classes	362	43.6
**School type**		
Public school	412	49.6
Private school	418	50.4
**Religion**		
Christian	630	75.9
Islam	200	24.1
**Tribe**		
Yoruba	668	80.5
Igbo	106	12.8
Hausa/other tribes	56	6.7
**Parents living together**		
No	73	8.8
Yes	757	91.2
**Present family setting**		
Extended/step/grandparent/single parent	176	21.2
Nuclear family	654	78.8
**Social class**		
Low social class	167	20.1
Middle social class	252	30.4
High social class	410	49.5

**Tobacco use status:** the prevalence of susceptibility to cigarette smoking and having the intention to smoke cigarette among the respondents were 25.9% and 6.3%, respectively (Table 2). About 88 (10.6%) and 102 (12.3%) had smoking friends and family members, respectively, while 100 (12%) said cigarette was sold near their school.

**Table 2:** tobacco use attitudes and habits of non-smoking school-going adolescents in Ibadan, Nigeria

Variables	Options	Frequency (830)	Percent (%)
It is safe to smoke tobacco for only a year or two as long as you quit after that	No	678	81.7
	Yes	152	18.3
Smoke from other people's tobacco smoking is harmful to you	No	182	21.9
	Yes	648	78.1
Smoking tobacco is harmful to your health	No	105	12.7
	Yes	725	87.3
Most people my age smoke tobacco	I believe this	468	56.4
	I don't believe this	162	19.5
	I don't know	200	24.1
Attitude to cigarette smoking in categories	Positive	178	21.4
	Negative	652	78.6
Susceptible to future cigarette uptake	No	615	74.1
	Yes	215	25.9
Smoking intention	No (no intention)	778	93.7
	Yes (intention)	52	6.3
Ever cigarette smoker	Yes	53	6.4
	No	777	93.6
Ever-smoker of tobacco products other than cigarette	Yes	49	5.9
	No	781	94.1
Ever-use of smokeless tobacco products such as snuff	Yes	13	1.6
	No	817	98.4
Close friends smoke	Yes	88	10.6
	No	742	89.4
Close family members smoke	Yes	102	12.3
	No	728	87.7
Number of days of exposure to SHS, inside your home, in your presence	Yes (1-7 days)	52	6.3
	Nil (0 days)	778	93.7
Number of days of exposure to SHS, inside any enclosed public space, other than your home	Yes (1-7 days)	169	20.4
	Nil (0 days)	661	79.6
Number of days of exposure to SHS, at any outdoor public place	Yes (1-7 days)	226	27.2
	Nil (0 days)	604	72.8
Sent to buy tobacco by elders	Yes	56	6.7
	No	774	93.3
Find it easy or difficult to buy cigarette from a shop	Easy	47	5.7
	Difficult	206	24.8
	Did not buy cigarettes	577	69.5
Cigarette sold near your school	Yes	100	12.0
	No/I don't know	730	88.0
Can purchase [tobacco/cigarettes] near your school	Yes	53	6.4
	No/I don't know	777	93.6
School has a policy against tobacco smoking inside school buildings	Yes	396	47.7
	No	152	18.3
	I don't know	282	34.0

**Factors associated with susceptibility and future intention to smoke cigarette:** factors such as having both parents living together, attitude towards cigarette smoking, ever-use of cigarette, other smoked and smokeless tobacco products were significantly associated with both susceptibility and future intention to smoke (p<0.005) (Table 3). Other factors were having smoking friends, tobacco sales near schools, difficulty in purchasing cigarettes from shops and the harm perceptions towards cigarette smoking, exposure to SHS and the safety of short-term smoking (p<0.05) (Table 3).

**Table 3:** factors associated with susceptibility and future intention to smoke cigarette among current non-cigarette smoking adolescents in Ibadan, Nigeria

Variables	Options	Susceptible to cigarette smoking		Future smoking intention	
		Yes (n=215)	p-value	Yes (n=52)	p-value
School type	Public	126 (30.6%)	0.002^*^	31 (7.5%)	0.137
	Private	89 (21.3%)		21 (5.0%)	
Parents living together	No	27 (37.0%)	0.024^*^	10 (13.7%)	0.006^*^
	Yes	188 (24.8%)		42 (5.5%)	
Present family setting	Extended	62 (35.2%)	0.001^*^	16 (9.1%)	0.081
	Nuclear	153 (23.4%)		36 (5.5%)	
Social class	Low	57 (34.1%)	0.002^*^	17 (10.2%)	0.065
	Middle	73 (29.0%)		14 (5.6%)	
	High	85 (20.7%)		21 (5.1%)	
Religion	Christianity	142 (22.5%)	<0.001^*^	38 (6.0%)	0.623
	Islam	73 (36.5%)		14 (7.0%)	
Sex	Male	124 (29.1%)	0.030^*^	32 (7.5%)	0.128
	Female	91 (22.5%)		20 (5.0%)	
Attitude to cigarette smoking	Negative	71 (39.9%)	<0.001^*^	19 (10.7%)	0.006^*^
	Positive	144 (22.1%)		33 (5.1%)	
Smoke from other people's tobacco smoking is harmful to you	No	89 (48.9%)	<0.001^*^	20 (11.0%)	0.003^*^
	Yes	126 (19.4%)		32 (4.9%)	
Smoking tobacco is harmful to your health	No	64 (61.0%)	<0.001^*^	12 (11.4%)	0.019^*^
	Yes	151 (20.8%)		40 (5.5%)	
Safe to smoke tobacco for 1-2 years as long as you quit after that	No	157 (23.2%)	<0.001^*^	33 (4.9%)	<0.001^*^
	Yes	58 (38.2%)		19 (12.5%)	
Ever cigarette smoker	Yes	23 (43.4%)	0.003^*^	43 (5.6%)	0.001^*^
	No	192 (24.7%)		9 (17.0%)	0.004^∞*^
Ever-smoker of tobacco products other than cigarette	Yes	30 (61.2%)	<0.001^*^	11 (22.4%)	<0.001^*^
	No	185 (23.7%)		41 (5.2%)	<0.001^∞*^
Ever-use of smokeless tobacco products such as snuff	Yes	7 (53.8%)	0.020^*^	4 (30.8%)	<0.001^*^
	No	208 (25.5%)	0.048^∞*^	48 (5.9%)	0.006^∞*^
Close friends smoke	Yes	32 (36.4%)	0.018^*^	12 (13.6%)	0.003^*^
	No	183 (24.7%)		40 (5.4%)	
Number of days of exposure to SHS, inside your home, in your presence	Yes (1-7 days)	21 (40.4%)	0.014^*^	3 (5.8%)	0.879
	Nil (0 days)	194 (24.9%)		49 (6.3%)	1.000^∞^
Sent to buy tobacco by elders	Yes	19 (33.9%)	0.156	9 (16.1%)	0.002^*^
	No	196 (25.3%)		43 (5.6%)	0.006^∞*^
Would find it easy or difficult to buy cigarette from a shop	Easy	24 (51.1%)	<0.001^*^	13 (27.7%)	<0.001^*^
	Difficult	67 (32.5%)		17 (8.3%)	
	Did not buy cigarettes	124 (21.5%)		22 (3.8%)	
Cigarette sold near your school	Yes	37 (37.0%)	0.007^*^	11 (11%)	0.037^*^
	No/I don't know	178 (24.4%)		41 (5.6%)	
Can purchase [tobacco/cigarettes] near your school	Yes	21 (39.6%)	0.018^*^	4 (7.5%)	0.691
	No/I don't know	194 (25.0%)		48 (6.2%)	0.567 ^∞^
The school have a policy or rule specifically prohibiting tobacco use among students inside school buildings	Yes	85 (21.5%)	0.003^*^	26 (6.6%)	0.255
	No	54 (35.5%)		13 (8.6%)	
	I don't know	76 (27.0%)		13 (4.6%)	

∞ : Fisher's exact test; ^*^significant

Following regression analysis, factors that were independently associated with adolescent´s susceptibility were low social class (aOR: 1.68; 95%CI: 1.01-2.80), poor attitude to smoking (supports smoking) (aOR: 1.93; 95%CI: 1.29-2.89), no/limited harm perception towards cigarette smoking (aOR: 3.55; 95%CI: 2.13-5.92) and exposure to SHS (aOR: 2.31; 95%CI: 1.52-3.50). Others are perceived safety of short-term smoking (aOR: 1.59; 95%CI: 1.02-2.44) and cigarette sale near the schools (aOR: 2.04; 95%CI: 1.16-3.61) (Table 4).

Predictors of adolescents´ future intention to smoke cigarette were ever-tobacco smoking (aOR: 2.05; 95%CI: 1.003-4.170), having a poor attitude to smoking (supports smoking) (aOR: 1.95; 95%CI: 1.31-2.88), no/limited harm perception towards cigarette smoking (aOR: 3.87; 95%CI: 2.38-6.31) and exposure to SHS (aOR: 2.45; 95%CI: 1.64-3.67). Others were perceived safety of short-term smoking (aOR: 1.59; 95%CI: 1.05-2.44) and cigarette sale near the schools (aOR: 1.79; 95%CI: 1.09-2.94) (Table 4).

**Table 4:** predictors of smoking susceptibility and future intention to smoke among current non-smoking, school-going adolescents in Ibadan, Nigeria

Variables	Susceptible to smoke cigarette			Future smoking intention		
	aOR	95% CI	p-value	aOR	95% CI	p-value
**Social class**						
Low social class	1.68	1.01-2.80	0.046^*^			
Middle social class	1.20	0.77-1.89	0.427			
High social class	1.00					
**Ever-smoker of tobacco products other than cigarette**						
Yes				2.05	1.003-4.170	0.049^*^
No				1.00		
**Attitude towards cigarette smoking**						
Support smoking/poor attitude/0-7	1.93	1.29-2.89	0.001^*^	1.95	1.31-2.88	0.001^*^
Against smoking/good attitude/8-16	1.00			1.00		
**Cigarette sold near your school**						
Yes	2.04	1.16-3.61	0.014^*^	1.79	1.09-2.94	0.022^*^
No/I don't know	1.00			1.00		
**Smoke from other people's tobacco smoking is harmful to you**						
Definitely/probably not	2.31	1.52-3.50	<0.001^*^	2.45	1.64-3.67	<0.001^*^
Probably/definitely yes	1.00			1.00		
**Smoking tobacco is harmful to your health**						
Definitely/probably not	3.55	2.13-5.92	<0.001^*^	3.87	2.38-6.31	<0.001^*^
Probably/definitely yes	1.00			1.00		
**It is safe to smoke tobacco for only a year or two as long as you quit after that**						
Definitely/probably yes	1.59	1.02-2.44	0.038^*^	1.59	1.05-2.44	0.029^*^
Probably/definitely not	1.00			1.00		

* Significant; 95%CI: 95% confidence interval; aOR: adjusted odds ratio

## Discussion

Susceptibility to tobacco smoking and intention to smoke are stages preceding the eventual experimentation or initiation of smoking. These stages are very important because when an individual is in this stage, he/she is more likely than others to eventually initiate the habit. Therefore, the importance of tobacco control efforts geared at reducing the prevalence of susceptible adolescents, as well as those who are nursing a future ambition to smoke cannot be overemphasized. This study was conducted among school-going adolescents in Ibadan, Nigeria and focused on those who were non-cigarette smokers at the time of data collection in 2018. The prevalence of susceptibility to cigarette smoking was found to be significantly high, such that one out of every four respondents were found to be susceptible while the proportion of those who had the intention to smoke a cigarette in the next 12 months was comparatively low (6.3%).

Nigeria has a very youthful population [43]. According to the 2016 estimate by the UNICEF, over 42 million (22%) of the country´s population were adolescents (10 - 19 years) [43]. Thus, with a prevalence of susceptibility to smoking being 25.9%, it suggests that the number of adolescents who are susceptible across the country may be very high. Also, this study was conducted among in-school adolescents, leaving out adolescents who are out of school. However, studies have reported that tobacco habit among out-of-school adolescents is often higher than among in-school adolescents [44,45], hence, the proportion of out of school adolescents who are susceptible to tobacco smoking may even be higher. This calls for more nationally representative studies to be conducted among both in-school and out-of-school adolescents.

The prevalence of smoking susceptibility among adolescents, recorded from this study is in line with reports from many other studies, most of which reported a high prevalence of susceptibility to smoking among adolescents, ranging between 13.2% and 49.7% [21-32,37]. Though the prevalence of those intending to smoke a cigarette in the next 12 months was a lot lower than that of those susceptible to smoking, the occurrence is still of concern. This is because, at 6.3%, it is twice the prevalence of the current cigarette smokers (3.6%) among the same study population. If all those intending to smoke in the next 12 months eventually initiate the habit, the smoking prevalence would have increased by over 200%. Therefore, tobacco intervention programmes aimed at preventing the eventual uptake of cigarette smoking among this group becomes very key.

The prevalence of susceptibility and intention to smoke from previous studies vary widely. Though the burden of tobacco use also varies widely across the globe, due to factors such as socio-cultural differences and the effectiveness of tobacco control legislations [23]; however, we believe that the different measures used for categorizing susceptibility and intention to smoke in these studies [21-32] are also an important factors that may influence the outcome measures. For example, the criteria used for classifying non-smoking status in these studies differ; while in some of the studies, it was restricted to never-smokers, some studies referred to only current non-smokers and other studies included all categories (current, experimenters and never smokers). Since these factors are independently associated with susceptibility and intention to smoke, they may have contributed to the disparities recorded. We suggest that authors should agree on a consensus on how to measure smoking susceptibility and future smoking intention, to allow for a more accurate comparison of data across countries. We suggest that the validated single question measure for future smoking intention [15] and a three-question measure for susceptibility to tobacco smoking [16], such as was used in this study be adopted.

Poor attitude (support for cigarette smoking) was a predictor of susceptibility and future intention to smoke cigarettes. This is not surprising as having a belief/attitude that supports a particular habit is usually a step towards practicing such behaviour. Our finding is in line with several other studies that associated attitude towards smoking with susceptibility to smoking [23,31,32] and future intention to smoke cigarette [35,46] in their reports. Furthermore, adolescents with no harm perception from cigarette smoking or exposure to SHS were more likely to be susceptible and have smoking intention. This finding is in agreement with other similar studies that reported that the two outcome variables were directly related to smoking-related perceptions of risks [20,24,31,32]. Over half of the respondents believed that most people of their age smoke. This perceived prevalence is high and should be a cause for concern. This is because the higher the perceived prevalence of a habit, the more likely it will be considered a norm, and the higher the chance that the person will initiate the habit [47]. These results have shown that tobacco prevention interventions should target adolescents' attitudes/perceptions of tobacco smoking.

The tobacco law in Nigeria banned the sale of tobacco products near schools, but this law has not been effective. Adebiyi *et al*. (2017) reported that 87% of the schools surveyed had tobacco products on sale within 100 meters of the school premises [48]. This action was found to be one of the predictors of future cigarette smoking in this study. As such those who had tobacco on sale near their schools being about two times more likely than those who do not, to be susceptible to smoking and having future intention to smoke cigarettes. The situation calls for the need to ensure the implementation of the related laws, as well as other tobacco control laws in Nigeria. Socio-economic status has not been reported by previous studies as a predictor for susceptibility to cigarette smoke, but we found in this study that those in the low social class were almost twice more likely than those in the high social class to be susceptible to smoking. We suggest that further studies be carried out to determine the role of SES on smoking susceptibility so that future tobacco intervention programmes are appropriately guided.

We found that those who ever smoked other tobacco products other than cigarettes were two times more likely than never-tobacco smokers to have the intention to smoke a cigarette in the next 12 months. This finding agrees with previous studies that had reported that smoking non-cigarette tobacco products, such as shisha, may eventually serve as the gateway to the initiation of cigarette smoking [49-51]. Similarly, this also underscores the importance of preventing adolescents from initiating the tobacco habit, since the experimenters, irrespective of the tobacco product used, are likely to have future cigarette smoking intentions [23,49,50].

There is a dearth of research on adolescents´ smoking susceptibility and future intention to smoke in Nigeria and this current study provides a valuable empirical insight into the prevalence and predictors of these behavioural traits. However, the study has its limitations, such as its inability to infer causality being a cross-sectional study; misreporting, since it is based on self-report; and limitation in generalizing the result for the whole country since it was among adolescents in one city, Ibadan. We recommend further studies from other parts of the country to investigate the possible determinants of susceptibility and future intention to smoke; so that adequate national data will be available to aid policy formulation and plan effective interventions.

## Conclusion

The prevalence of susceptibility to cigarette smoking and future intention to smoke among current non-smoking, school-going adolescents is high. Some of the predictors include having a poor attitude towards smoking, belief that the harm from cigarette and its smoke is low as well as the sale of tobacco products close to schools.

### What is known about this topic

- Tobacco use remains the leading cause of preventable death among both the users and the non-users that were exposed to the smoke;
- Eighty percent of adult smokers picked up smoking habits before the age of 18 and half of them suffer tobacco-related death and illnesses.

### What this study adds

- The prevalence of susceptibility to smoking among school-going adolescents appears to be increasing when compared to previous studies while that of future intention to smoke is significantly high;
- The proportion of school-going adolescents who intend to smoke cigarettes in the future is greater than that of the current smokers among the same group;
- Adolescents´ social class is independently associated with their susceptibility to cigarette smoking and this had not been previously reported.
